# Asymmetric cross-hemispheric connections link the rat anterior thalamic nuclei with the cortex and hippocampal formation

**DOI:** 10.1016/j.neuroscience.2017.02.026

**Published:** 2017-05-04

**Authors:** Mathias L. Mathiasen, Christopher M. Dillingham, Lisa Kinnavane, Anna L. Powell, John P. Aggleton

**Affiliations:** a School of Psychology, Cardiff University, Tower Building, 70 Park Place, Cardiff CF10 3AT, UK; b Trinity College Institute of Neuroscience, Trinity College Dublin, Dublin, Ireland

**Keywords:** AD, anterodorsal thalamic nucleus, AM, anteromedial thalamic nucleus, a-p, anterior-posterior, AV, anteroventral thalamic nucleus, BDA, biotinylated dextran amine, Cg, cingulate cortex, DY, diamidino yellow, FB, fast blue tracer, FG, fluorogold tracer, HPC, hippocampus, IAD, interanterodorsal thalamic nucleus, IAM, interanteromedial thalamic nucleus, LD, laterodorsal thalamic nucleus, LP, lateral posterior thalamic nucleus, M2, secondary motor cortex, MD, mediodorsal thalamic nucleus, PL, prelimbic cortex, PoS, postsubiculum, PT, parataenial thalamic nucleus, RSC, retrosplenial cortex, RSD, dysgranular retrosplenial cortex, RSG, granular retrosplenial cortex, Sm, stria medullaris of the thalamus, SUB, subiculum, V1, primary visual cortex, V2, secondary visual cortex, VA, ventral anterior thalamic nucleus, VL, ventrolateral thalamic nucleus, WGA-HRP, horseradish peroxidase-conjugated wheat germ agglutinin, Contralateral, Corticothalamic, Hippocampus, Interhemispheric, Thalamocortical, Thalamus

## Abstract

Dense reciprocal connections link the rat anterior thalamic nuclei with the prelimbic, anterior cingulate and retrosplenial cortices, as well as with the subiculum and postsubiculum. The present study compared the ipsilateral thalamic-cortical connections with the corresponding crossed, contralateral connections between these same sets of regions. All efferents from the anteromedial thalamic nucleus to the cortex, as well as those to the subiculum, remained ipsilateral. In contrast, all of these target sites provided reciprocal, bilateral projections to the anteromedial nucleus. While the anteroventral thalamic nucleus often shared this same asymmetric pattern of cortical connections, it received relatively fewer crossed inputs than the anteromedial nucleus. This difference was most marked for the anterior cingulate projections, as those to the anteroventral nucleus remained almost entirely ipsilateral. Unlike the anteromedial nucleus, the anteroventral nucleus also appeared to provide a restricted, crossed projection to the contralateral retrosplenial cortex. Meanwhile, the closely related laterodorsal thalamic nucleus had almost exclusively ipsilateral efferent and afferent cortical connections. Likewise, within the hippocampus, the postsubiculum seemingly had only ipsilateral efferent and afferent connections with the anterior thalamic and laterodorsal nuclei. While the bilateral cortical projections to the anterior thalamic nuclei originated predominantly from layer VI, the accompanying sparse projections from layer V largely gave rise to ipsilateral thalamic inputs. In testing a potentially unifying principle of anterior thalamic – cortical interactions, a slightly more individual pattern emerged that reinforces other evidence of functional differences within the anterior thalamic and also helps to explain the consequences of unilateral interventions involving these nuclei.

## Highlights

• Thalamocortical projections from ATN are ipsilateral with the exception of a restricted bilateral AV projection to RSC.
• Corticothalamic projections to the ATN are bilateral with the exception of an ipsilateral Cg projection to the AV nucleus.
• The subiculum receives ipsilateral ATN efferents and provides the ATN with bilateral afferents.
• The LD nucleus has exclusively ipsilateral, bidirectional connections with investigated cortical and hippocampal targets.
• The postsubiculum has exclusively ipsilateral, bidirectional connections with the ATN, as well as the LD nucleus.

## Introduction

Reciprocal connections between the thalamus and cortex underlie numerous brain functions. Attention has naturally focussed on the dense, ipsilateral thalamocortical projections, which are often complemented by return thalamic inputs from deep cortical layers (Deschenes et al., 1998, Sherman, 2007). In addition to these ipsilateral connections, there are both indirect and direct pathways that provide inter-hemispheric communication between the thalamus and contralateral cortex. One indirect pathway involves the commissural fibers that connect corresponding cortical areas across the two hemispheres. There are, in addition, direct connections that link the thalamus with the cortex in the opposite hemisphere. Their prevalence remains uncertain, however, as the lack of a description of a particular crossed thalamic-cortical connection does not confirm its absence, with many papers leaving this information unspecified.

The current study focussed on the rat anterior thalamic nuclei (ATN). The ATN comprise three principal nuclei, the anteromedial (AM), anteroventral (AV), and anterodorsal (AD) nucleus, along with a fourth nucleus in the rodent, the interanteromedial nucleus (IAM) (Swanson, 1992). We also included the laterodorsal (LD) nucleus as it displays clear hodological similarities with the ATN (van Groen and Wyss, 1990a, van Groen and Wyss, 1992b, Shibata, 1996, Shibata, 2000, van Groen et al., 2002b). These combined nuclei have important roles in human episodic memory and rodent spatial memory (Sutherland and Rodriguez, 1989, Aggleton and Sahgal, 1993, Taube, 1995, Byatt and Dalrymple-Alford, 1996, Aggleton and Brown, 1999, Harding et al., 2000, Vertes et al., 2001, Warburton et al., 2001, van Groen et al., 2002a, Aggleton et al., 2010, Clark and Taube, 2012, Jankowski et al., 2013).

The ATN have numerous reciprocal connections with the cortex and hippocampal formation, the latter involving subicular regions (van Groen and Wyss, 1990a, van Groen and Wyss, 1990b, Shibata, 1993, Shibata and Kato, 1993, van Groen et al., 1999, Van Groen and Wyss, 2003, Shibata and Naito, 2005, Wright et al., 2010). Where it has been specified, ATN projections to rat frontal and cingulate areas remain ipsilateral, while the corresponding ATN afferents more often appear bilateral in origin (Dekker and Kuypers, 1976, Swanson and Cowan, 1977, Kaitz and Robertson, 1981, Seki and Zyo, 1984, Oda, 1997, Shibata and Naito, 2005). Nonetheless, weak anterior thalamic projections to the contralateral cortex have been described in primates (Dermon and Barbas, 1994), suggesting it would be premature to exclude the possibility of such projections in the rat. There is also disagreement over whether all cortical projections to the rat AM and AV nuclei are bilateral (Seki and Zyo, 1984, Oda, 1997, Shibata and Naito, 2005).

Both anterograde and retrograde tracers helped to determine whether the apparently asymmetric pattern of bilateral (thalamic afferents) and ipsilateral (thalamic efferents) connections is a general feature of the anterior and laterodorsal thalamic nuclei. We also analyzed the laminar origin of the corticothalamic projections to ATN in both hemispheres in light of current ideas about lamina distinctions (Sherman, 2016). In addition to neocortical regions, we also analyzed the reciprocal ATN connections with the subiculum and postsubiculum, in light of the importance of the hippocampal formation for ATN function.

## Experimental procedures

### Animals

A total of 32 adult male Lister Hooded rats (weight 290-350 g, Harlan Laboratories, United Kingdom) were examined for this study. All procedures were approved by the appropriate ethics committee at Cardiff University and followed the UK Animals (Scientific Procedures) Act (1986).

### Surgical procedures

All surgeries were performed under an isoflurane-oxygen mixture. The animals were first anesthetized and then placed in a stereotaxic frame. The tracer injection coordinates were partly guided by a stereotaxic brain atlas (Swanson, 1992), corrected by the weight of the animal as well as by comparisons with previous tracer injections.

### Choice of tracer

A total of 37 neuroanatomical tracer injections were analyzed as we injected two tracers in five of the animals (Table 1). Typically in the cortex, we used the anterograde tracer *biotinylated dextran amine* (BDA) to map corticothalamic pathways and the retrograde tracer *fast blue* (FB) to map the thalamocortical pathways. In one case the retrograde tracer *fluorogold* (FG) was used since that particular injection (in postsubiculum) required the tracer deposit to cover a larger area (which from our experience is a feature of FG). The 3 kD version of BDA was predominantly used in this study, which can also be transported in the retrograde direction (Fritzsch, 1993, Kaneko et al., 1996, Medina et al., 1997). This retrograde component of BDA is not as strong as that seen in *horseradish peroxidase conjugated wheat germ agglutinin* (WGA-HRP) (Gonatas et al., 1979, Mesulam and Brushart, 1979). Therefore, in two regions (the prelimbic cortex and the subiculum) that were not sufficiently covered by FB injections, we used WGA-HRP to enhance the retrograde component as well as to provide an anterograde signal. Survival times for WGA-HRP injections were kept relatively short (2 days) in order to limit the risk of trans-synaptic transport (Itaya and van Hoesen, 1982, Itaya et al., 1986, Itaya, 1987).

Additional data came from injecting the retrograde tracers *fast blue* (FB) and *diamidino yellow* (DY) into the AV, AM and LD thalamic nuclei. The tracers FB and DY, which are transported exclusively in the retrograde direction, produce largely unambiguous labeling of the cell bodies (Bentivoglio et al., 1980, Conde, 1987, Byers et al., 2002). These tracers helped to validate the results obtained from the BDA injections in cortex. In a few cases, WGA-HRP and BDA were injected into the ATN but, in these cases only, the anterograde component was analyzed (see Result section for further clarification).

### Tracer injections and visualisation

All injections, except one (see below), were made via a 0.5-μl or 1-μl Hamilton syringe (Hamilton, Bonaduz, Switzerland). Both FB (Polysciences Inc, Eppelheim, Germany) and DY (Sigma–Aldrich, Gillingham, United Kingdom) were made up as a 3% solution in sterile phosphate-buffered saline (PBS; pH 7.4). Fluorogold was made up as a 4% solution in double distilled water (Santa Cruz biotechnology, US). WGA-HRP (Vector Labs, Peterborough, UK) was used at a concentration of 40 mg/ml, while 3 kD BDA (Life Technologies Ltd, Paisley, UK) was made up at 10% in sterile, distilled water (pH 7.4). Typically, individual injection volumes at each thalamic site were 0.04–0.06 µl and 0.06–0.08 µl for each cortical site. In most cases, tracer injections into the neocortex involved multiple anterior-posterior levels. In one case, we iontophoretically injected the 10 kD version of BDA (Invitrogen, United Kingdom) into a single site in the anterior cingulate cortex via a glass micropipette (18–22-µm tip diameter). In this case we used an alternating current (6 s on/off) of 6 µA in 10 m. In all other cases, tracers were delivered by pressure injections made over the course of 10 min with the needle left *in situ* prior to injection for 5 min. Following surgery, animals recovered in a thermostatically controlled container before being returned to individual housing with *ad libitum* food and water.

Following a tracer dependent, post-operative period of 2–7 days, the animals were deeply anesthetized with sodium pentobarbital (Euthatal, Merial, Harlow, UK) and perfused transcardially with 0.1 M PBS (pH 7.4) at room temperature followed by 4% paraformaldehyde in 0.1 M PBS (pH 7.4). In those animals that received injections of WGA-HRP, the fixative was composed of 1.5% paraformaldehyde and 1.5–2% glutaraldehyde in 0.1 M PBS. Brains were removed and placed in the dark for 4 h in fixative and then transferred to a 25% sucrose solution in 0.1 M PBS for 24 h in the dark to cryoprotect the tissue before cutting. Brains were placed on a freezing platform and 40-µm coronal sections cut on a sledge microtome (Leica 1400) in four series. For those cases with fluorescent tracers, two series of sections were mounted directly onto gelatine-subbed slides and then allowed to dry in the dark at room temperature. One series was stained with cresyl violet.

In cases with BDA or WGA-HRP injections, one series was mounted directly onto gelatine-subbed slides, for subsequent cresyl violet staining. The remaining series were collected in 0.1 M phosphate puffer. For brains with WGA-HRP injections, these later series were processed with 3,3′5,5′ tetramethylbenzidine (TMB). For the TMB reaction, sections were incubated, with agitation, in a fresh 0.1 M phosphate buffer (PBS, pH 6.0) solution before being transferred to a solution containing 0.25% ammonium molybdate in 0.1 M PBS and 0.002% 3′3′5′5 tetramethylbenzidine, dissolved in 100% ethanol, again with agitation, for 30 min. Following incubation, a 1% hydrogen peroxide solution in distilled water was added in three stages with 30-min intervals until the final concentration of hydrogen peroxide was 0.3%. Sections were then incubated in the same solution overnight at 4 °C. The TMB reaction precipitate was stabilized through subsequent incubation of sections in a 5% ammonium molybdate solution in 0.1 M PBS (pH 6.0) for 30 min. Following incubation, sections were washed in 0.1 M PBS (pH 6.0) before being mounted on gelatin subbed slides and left to dry overnight at room temperature. The sections were then mounted and coverslipped with DPX mountant (Sigma).

In some cases the TMB method was supplemented with an immunohistochemical staining procedure, in which an antiserum directed against the WGA-HRP (Vector Labs) was used at a dilution of 1:2000 and incubated at 4 °C for 48 h. The antigen–antibody complex was localised with a standard avidin–biotin process (ABC Elite Kit, Vector Labs). The chromagen diaminobenzidine produced the visualised reaction product. The same procedure was used in cases with BDA tracer deposits, except that in these cases the biotinylated tracer was visualised without the use of antibodies. In some cases, instead of the ABC method, BDA was visualised by fluorophore (A488) conjugated streptavidin (Thermofisher, UK). In these cases the sections were washed 10 × 3 min in PBS (pH 7.4), incubated for 2 h at room temperature in PBS (1:200) (pH 7.4) and washed (10 × 30 min in PBS, followed by 5 × 10 m TNS, pH 7.4 for both). Reacted sections were then mounted onto gelatine-subbed processed as above.

A Leica DM5000B microscope with a Leica DFC310FX digital camera and Leica Application Suite image acquisition software were used for brightfield, darkfield and fluorescence microscopy. For some of the fluorescent images used in the figures, the contrast was improved following global modifications to hue and saturation. For some images, color information was converted to grey-scale.

In order to estimate the relative strength of the contralateral projections to the thalamus, compared to the corresponding ipsilateral projections, labeled cell counts were made in those cases with retrograde tracer deposits restricted to the ATN. Cell counts were made at equal intervals along the anterior-posterior (a-p) axis of the cortex and the hippocampal formation, although in three cases this analysis was restricted the analysis to limited portions of the a-p axis. In these three cases, bleaching of the fluorescence signal led to a reliance on previously acquired digital images to fully separate different tracer signals. The cell counts were made manually from the image software. Consequently, the counts were not intended to be stereological as the goal was to compare relative numbers across the same sites.

## Results

The anatomical designations and borders of the thalamic nuclei, as well as the various cortical and hippocampal regions, match those of Swanson (1992). Given its midline location, the IAM nucleus of the ATN is largely absent from the present descriptions although attention was given to the pattern of label it contained close to the border with AM, i.e., away from the midline.

### Cortex to the anterior thalamus

#### Retrosplenial cortex efferents (anterograde tracer injections)

In three cases, multiple injections of BDA (3kD) were made along the a-p axis of the retrosplenial cortex. The injections were centered in, but not exclusive to, layers II-V (186#4) or layers V (188#5) and V/VI (187#9) of the right hemisphere. All three BDA cases were centered in the granular retrosplenial cortex with either dense (188#5) or weak (187#9, 186#4) involvement of the dysgranular portion.

**Fig. 1:**
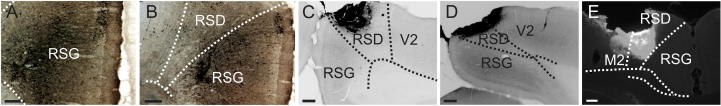
Photomicrographs showing the injection sites in three representative cases. The resulting label in all of these cases is illustrated in other figures. (A, B) Brightfield photomicrographs of the retrosplenial BDA injection in case 187#9. (C, D) Inverted grayscale fluorescence pictures of the retrosplenial fast blue tracer injections in cases 172#28 (C) and 172#27 (D). (E) Grayscale fluorescence picture of the retrosplenial fast blue injection in case 64#3. Dotted lines indicate borders between cortical regions. Scale bars = 200 µm.

**Fig. 2:**
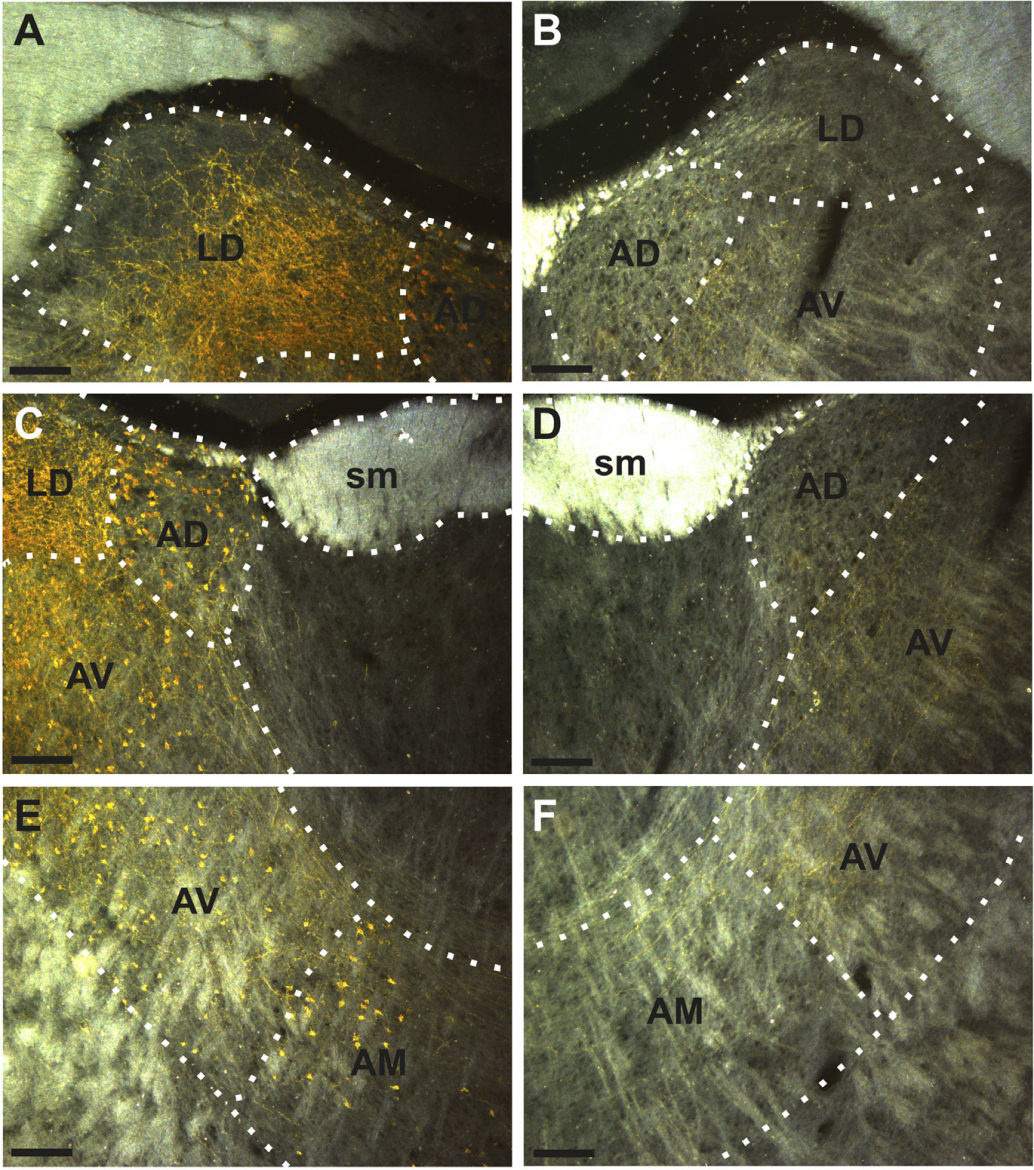
Darkfield photomicrograph illustrating the ipsilateral (A, C, E) and contralateral (B, D, F) fiber and cell distribution resulting from a BDA injection in the retrosplenial cortex (case 187#9). The injection site is shown in Fig.1A and B. All pictures are from the same coronal section at different dorsoventral levels. Scale bars = 150 µm.

In the two cases with injections centered in the deep cortical layers (188#5, 187#9; see Fig.1A,B) anterogradely labeled fibers were observed bilaterally in both AV and AM (Fig.2C-F, case 187#9). In both cases, the fiber label in AV was dense in both hemispheres whereas the AM fiber label was more moderate. In one of these cases, the contralateral AM label was very sparse (188#5). In both cases, only very sparse fiber labeling was seen in the contralateral AD nucleus, despite a relatively strong signal in the ipsilateral AD nucleus (Fig.2A-D). Likewise, a dense fiber plexus could be seen in ipsilateral LD, yet there were just a few single, contralateral fibers (Fig.2A,B).

In the third case (186#4), dense labeling was seen in the ipsilateral AM, AV and LD nuclei. However, in this same case with limited layer VI involvement, no contralateral label was seen in these nuclei. Only extremely scattered and sparse fiber labeling was present in the ipsilateral AD nucleus.

#### Medial frontal cortex efferents (anterograde tracer injections)

Two cases had WGA-HRP injections largely restricted to the rostral prelimbic cortex. In one case, the injection was centered in layers V/VI (88#1) whereas in the other case the core of the injection site was in layer III and the superficial portion of layer V (88#2). In the former case, where the tracer included both deep layers, dense bilateral anterograde label was present in the AM nucleus. In the second case, however, no labelled fibers were seen in ATN in either hemisphere.

**Fig. 3:**
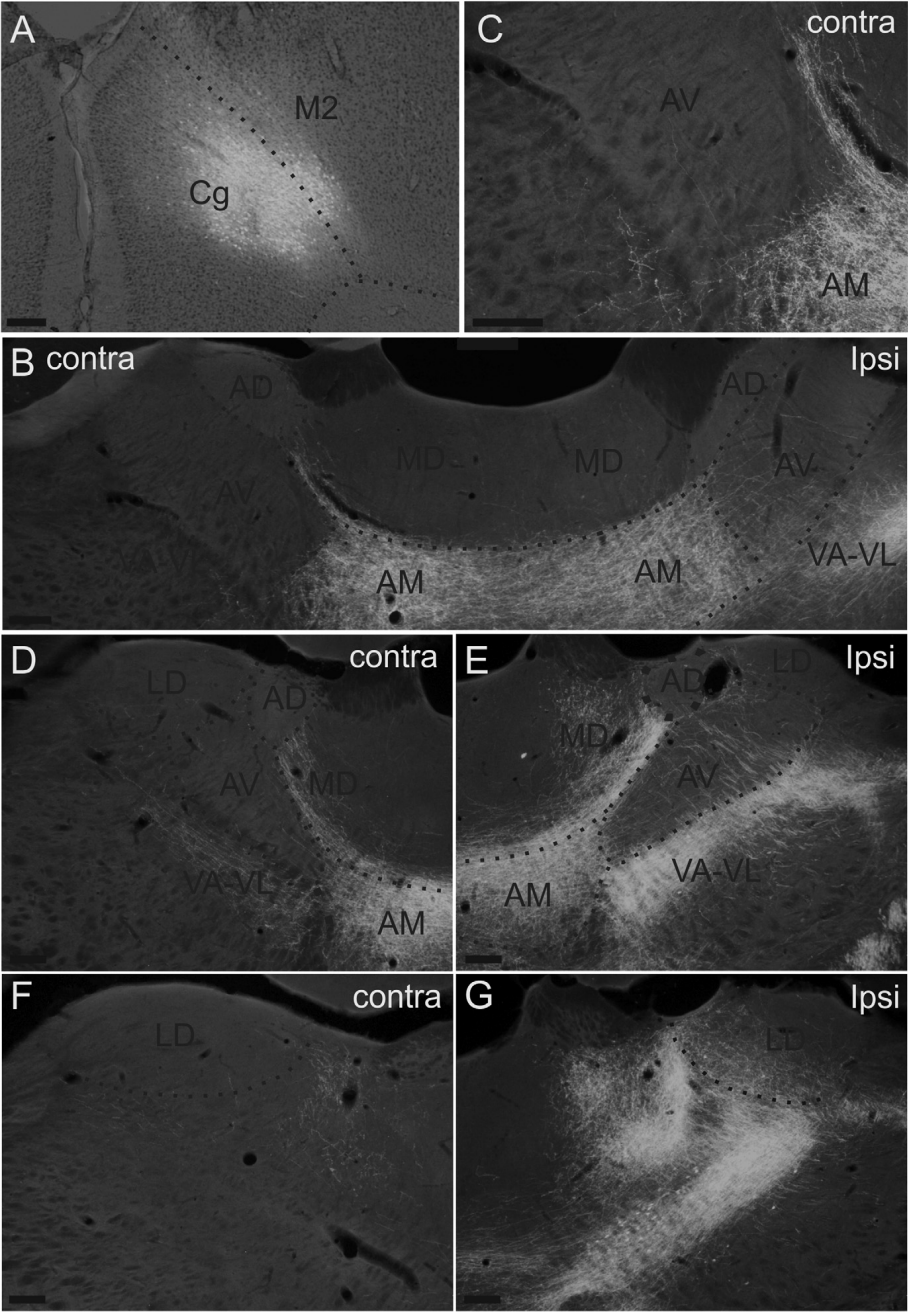
Fluorescence photomicrographs of anterograde label in the laterodorsal and anterior thalamic nuclei after a BDA injection into the anterior cingulate cortex (199#10). All pictures are converted to grayscale. (A) Photomicrograph of the BDA injection site. The image is an overlay of the fluorescence photomicrograph with its corresponding cresyl stained section. (B) BDA labeled fibers in the anterior thalamic nuclei at rostral levels. (C) A higher-resolution picture of the contralateral hemisphere in figure B. Figures D, E, F, G BDA labeled fibers in the laterodorsal and the anterior thalamic nuclei in the ipsilateral (E, G) and the contralateral (D, F) hemispheres. Scale bars = 200 µm.

In three cases (199#9, 199#10, 199#11), BDA was injected into the anterior cingulate cortex. In two of these cases the BDA was injected into all layers of the anterior cingulate cortex at various anterior-posterior levels, thereby covering a large extent of anterior cingulate cortex (199#9, 199#10). In both of these cases, the injection site, which involved a small portion of the secondary motor cortex, resulted in dense bilateral fiber labeling in AM (case 199#10, Fig.3B,D,E). In contrast, AV received a much more modest input that was virtually restricted to the ipsilateral hemisphere, with only extremely few scattered fibers in the contralateral hemisphere (Fig.3B,D,E). In LD, a moderate-to-dense fiber plexus was restricted to the ipsilateral hemisphere (Fig.3F,G).

In the final case (199#11), a smaller iontophoretic injection of BDA (10 kD) was restricted to layers III-VI of the anterior cingulate cortex with no involvement of other cortical areas. In this case, dense fiber innervation was seen bilaterally in AM but with only extremely sparse (ipsilateral only) AV and LD labeling. In all three cases, any AD labeling was extremely sparse and overwhelmingly in the ipsilateral hemisphere.

#### Subiculum efferents (anterograde tracer injections)

Anterograde tracer injections were placed in the dorsal subiculum in three cases (Table 1). In two cases (182#3, 182#4) BDA deposits were centered in a distal portion of the dorsal subiculum, although with potential weak tracer uptake in the overlying visual cortices (both cases) as well as in the postsubiculum (case 182#3). Another case had a WGA-HRP injection in a more proximal portion of the dorsal subiculum, and in this case (case 82#2) the tracer injection extended across into the distal CA1/proximal subicular border, with the weaker involvement of other hippocampal fields, reaching the ventral subiculum.

In the two cases with the BDA tracer injections more restricted to the subicular region (182#3, 182#4), ipsilateral fiber labeling was present in LD (both cases) while bilateral fiber labeling was seen in AM and AV. In the case with the greatest tracer involvement of CA1 (82#2), labeling was seen exclusively ipsilateral in LD, AD and AV.

#### Inputs to the anterior thalamic nuclei (retrograde tracer injections)

**Fig. 4:**
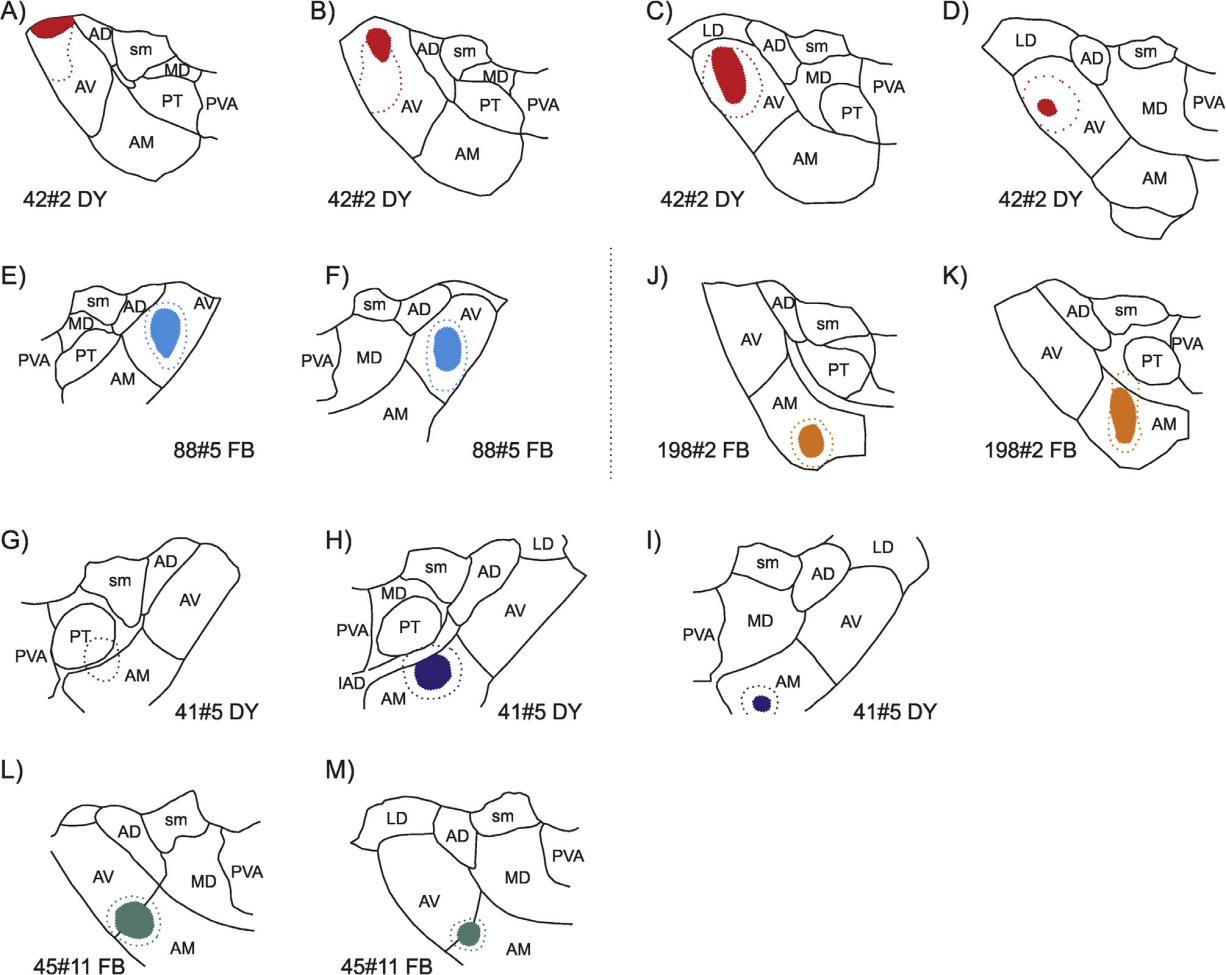
Line drawings of the retrograde injections restricted to the ATN. The filled area indicates the core of the injection site. The core is usually surrounded by an external “halo” with weaker intensity and this area is marked by the outer stippled line. (A-D) Plot of the diamidino yellow injection site in case 42#2. In this case the injection site is located in AV with no involvement of other thalamic nuclei. (E, F) Plot of the fast blue injection site in case 88#5. Again, the injection site is restricted to the AV nucleus. (J, K) Plot of the fast blue injection site in case 198#2. The injection site is centered in AM with weak involvement of the IAD and hippocampus (not shown). (G-I) Plot of the diamidino yellow injection site in case 41#5. Again, the injection site is restricted to AM except for a very weak potential involvement of the PT nucleus in the halo of the injection site. (L, M) Plot of the fast blue injection site in case 45#11. In this case the injection site involves both the AV and AM nuclei.

In order to test the results from our anterograde tracer injections into cortex (described above) we analyzed four cases with retrograde tracer injections (FB and DY) in either AV (88#5, 42#2 DY), AM (41#5 DY), or both nuclei (45#11 FB) (Fig. 4). Some of the thalamic nuclei adjoining these ATN nuclei also receive cortical inputs (see Fig. 3) and, hence, even the slight involvement of a nearby thalamic nucleus might produce misleading results. Accordingly, in the four cases selected the tracer deposits were almost entirely restricted to ATN, except for a very weak extension of the outer weak “halo” of the DY injection into the parataenial nucleus in case 41#5 (Fig.4G,H) (See below). We also included a fifth case with the tracer deposit centered in the AM nucleus (198#2). In this case, however, leakage along the injection tract resulted in weak tracer deposits in very restricted parts of the dentate gyrus and CA3, as well as in that portion of the ATN immediately dorsal to the AM nucleus (the interanterodorsal thalamic nucleus of Swanson (1992)) (Figs. 4J,K, 6A,B).

**Fig. 5:**
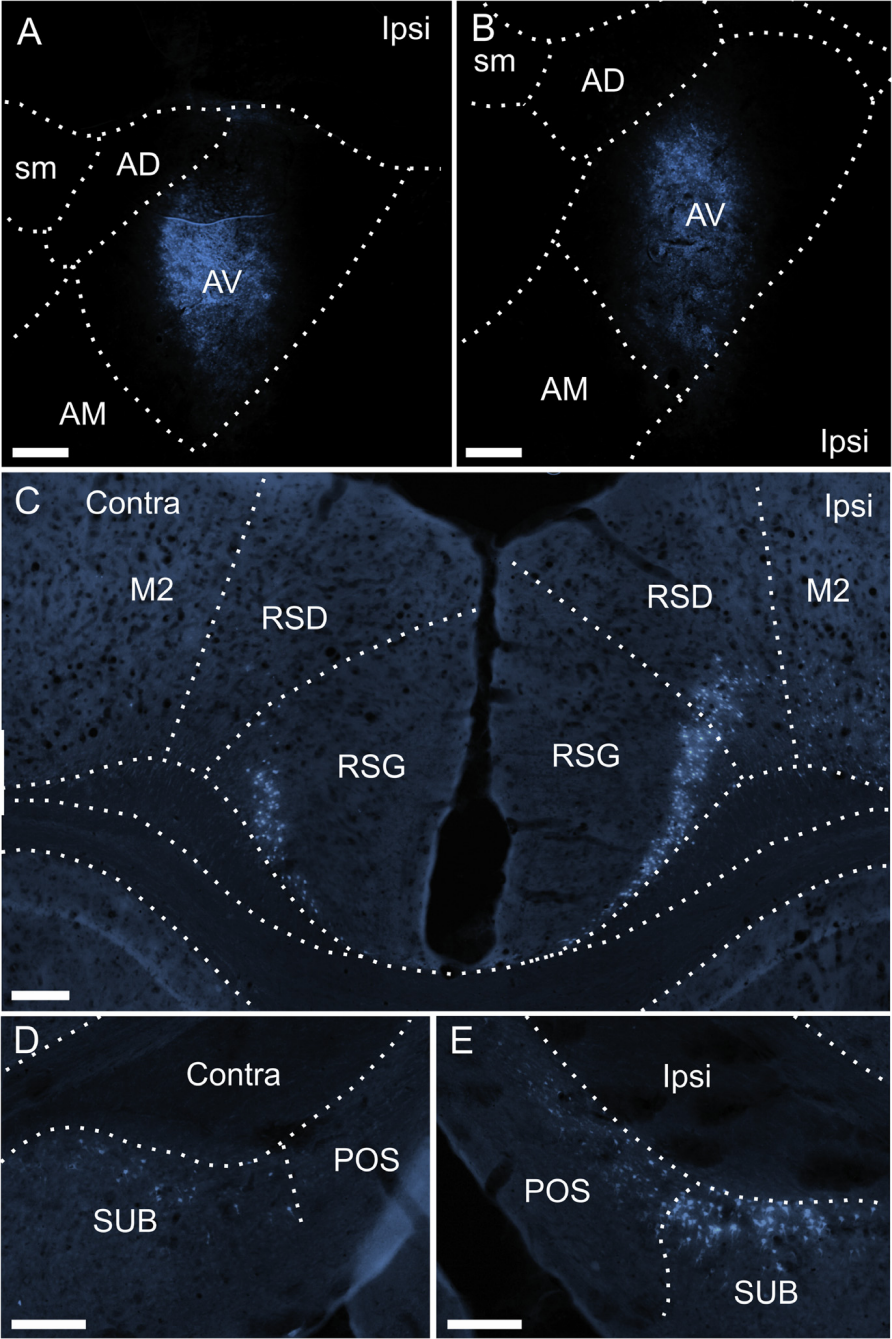
Fluorescence photomicrographs of retrograde labeled cells resulting from a fast blue injection restricted to the AV thalamic nucleus (Case 88#5). (A, B) Location of the fast blue deposit in the AV nucleus (see also Fig.4E,F). (C) bilateral cell labeling in the retrosplenial cortex. In this section there is appreciable bilateral label in granular retrosplenial cortex with only ipsilateral label in the dysgranular retrosplenial cortex (though sparse labeling was often present in the contralateral dysgranular field in other sections in this case). (D-E) Bilateral cell labeling in the subiculum in the contralateral (D) and ipsilateral (E) hemispheres. The label in the postsubiculum is restricted to the ipsilateral hemisphere. Scale bars = 200 µm.

In the two cases with tracers placed in AV, consistent bilateral labeling was observed in the subiculum and retrosplenial cortex (Figs. 5C,D,E, 11A-D). In the retrosplenial cortex, this bilateral label was generally most dense in the granular portion, whereas in the dysgranular portion the dense ipsilateral cell labeling was accompanied by sparser contralateral labeling (in some sections no contralateral thalamic labeling, e.g., Fig.5C). In contrast to the cases with AM injections (see below), the dense ipsilateral labeling in the anterior cingulate cortex was accompanied by either none (42#2 DY) or extremely sparse (88#5) labeling in the contralateral hemisphere. Furthermore, in these two cases, labeling in the prelimbic cortex was limited to either none (42#2 DY) or just a few labeled cells (all in the ipsilateral hemisphere) (88#5).

**Fig. 6:**
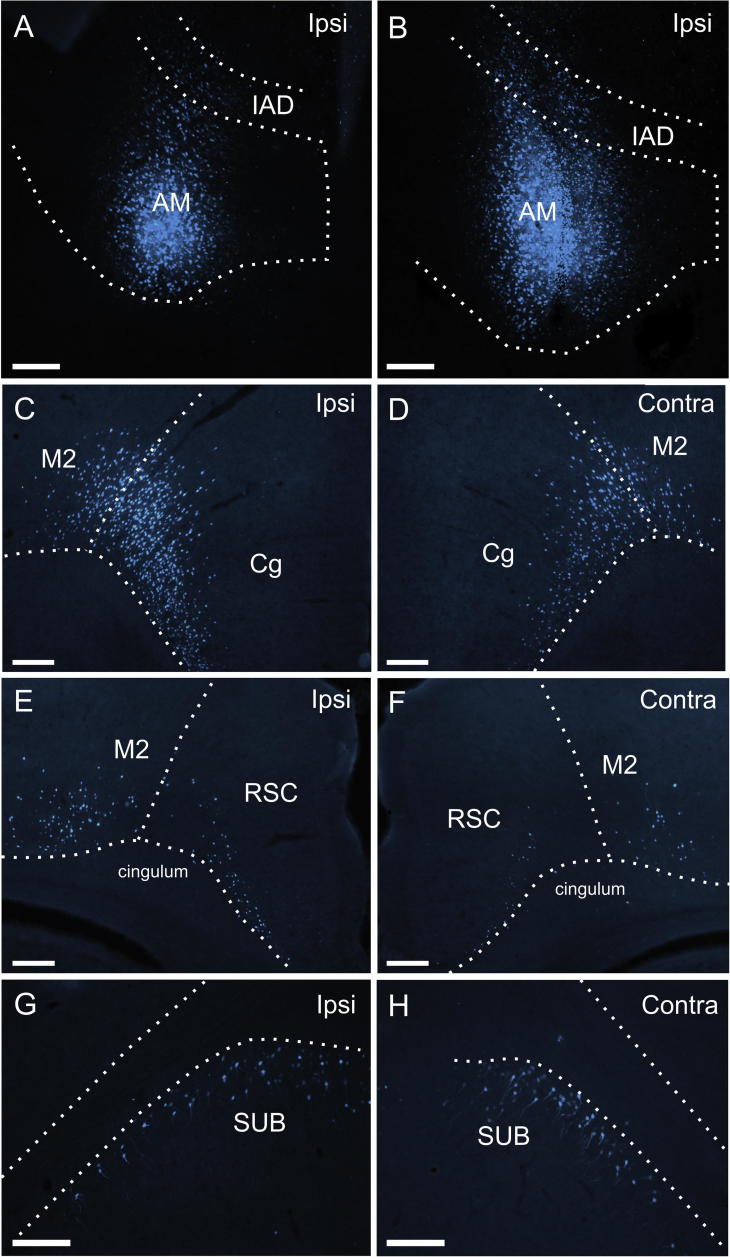
Fluorescence photomicrographs of retrograde labeled cells resulting from a fast blue injection centered in AM (Case 198#2, Fig.4J, K). Above the core of the fast blue injection, which is centered in AM, there is weak tract labeling in IAD that reaches the hippocampal formation (not shown). (A, B) The fast blue tracer deposit in AM, both pictures are from the same hemisphere. (C, D) Bilateral cell label in the ipsilateral (C) and contralateral (D) anterior cingulate cortex and adjacent M2. (E, F) Bilateral cell label in the ipsilateral (E) and contralateral (F) retrosplenial cortex and adjacent M2. (G, H) Bilateral label in the ipsilateral (G) and contralateral (H) subiculum. Scale bars = 200 µm.

In contrast to these two cases, when the injection site involved both AM and AV (45#11 FB) relatively dense bilateral label was present in the anterior cingulate cortex. This labeling pattern was also seen in the case 198#2 where the tracer deposits were centered in AM (see above) (Fig.6A,B). In these two cases, clear bilateral labeling was also present in the subiculum, as well as the prelimbic and retrosplenial cortices (Figs. 6, 11E-H). In the retrosplenial cortex, cells were observed bilaterally in both the granular and dysgranular portions, although dense labeling was only present in the granular portion (bilateral). In case 41#5 DY where the injection was restricted to AM, but positioned more caudal than in case 198#2, we only observed labeling in the anterior cingulate cortex, but again in both hemispheres.

In all of the cases with retrograde tracer injections in the ATN, a consistent laminar distribution of labeled cells was seen. In all neocortical regions analyzed, the dense plexus of labeled cells was found in layer VI with occasional sparse labeling of cells in layer V. The sparsely labeled cells in layer V were almost completely confined to the ipsilateral hemisphere. Consequently, the contralateral label (when present) was virtually restricted to layer VI in all cortical regions.

In stark contrast to all the above mentioned regions, the (rather weak) labeling in the postsubiculum remained restricted to the ipsilateral hemisphere (Fig.5D,E). To further test this negative finding, we also analyzed those retrograde tracer injections that were centered in the ATN but where there was some extension of the injection site into a nearby thalamic nucleus (88#6, 41#5 FB, 45#11 DY, 42#2 FB, 191#9, 198#4) (see Table 1). In all five cases, whenever cell labeling was present in the postsubiculum it was almost completely restricted to the ipsilateral hemisphere with only extremely few, occasional labeled cells in the contralateral hemisphere.

#### Inputs to the laterodorsal nucleus (retrograde tracer injections)

Two injections were centered in LD, although in both cases the tracer deposit reached restricted portions of either the VA-VL (196#19) or the LP (191#10) thalamic nuclei. In these cases, cell labeling was exclusively ipsilateral in the postsubiculum. There was also an overwhelmingly ipsilateral predominance of label in the subiculum, as well as the retrosplenial and anterior cingulate cortices.

#### Cell counts

We counted retrograde labeled cells in those cases with retrograde tracers restricted to the ATN nuclei. In the two cases with bilateral prelimbic label that predominantly originated from the AM nucleus (45#11; 198#2), the overall proportions of contralateral to ipsilateral cells were found to be 25.8% and 29.4%, respectively. When the tracer injections were increasingly restricted to AM (41#5; 198#2), the proportion of contra- to ipsilateral cells at rostral levels of the anterior cingulate cortex was substantially higher, reaching 90.2% and 77.0%, respectively. Although these numbers decreased when cells were counted along the entire a-p axis of the anterior cingulate cortex, the total proportion of contra- to ipsilateral cells (53.9%) was still substantially higher than that in the prelimbic cortex (case 198#2). These AM-related numbers contrasted with the almost complete lack of contralateral label in cases with restricted AV injections (42#2; 88#5). In agreement with this nuclear difference, in case 45#11 where the tracer deposit included both AM and AV (with the center of the injection site in AV) the proportion of contralateral anterior cingulate label (11.8%) was substantially reduced when compared to the above AM cases.

In the two cases where we counted along the full a-p extent of both the subiculum and the retrosplenial cortex the injections were restricted to either AM (198#2) or AV (88#5), thereby allowing for a direct comparison between the two nuclei. Based on these cases, there is a markedly higher proportion of contra- to ipsilateral cells projecting to AM than AV. Specifically, in retrosplenial cortex this proportion was 58.4% for projections to AM, compared to 33.5% for AV inputs. In the subiculum, the corresponding numbers are 72.0% for AM and 46.2% for AV. In two additional cases for analysis, which contained either a restricted AV injection (42#2) or an injection involving both AV and AM (45#11), the relative contralateral cell numbers were less than those in the selective AM case (198#2, see above) [for subiculum 22.2% (42#2) and 35.6% (45#11); for retrosplenial cortex 8.6% (42#2) and 14.8% (45#11)].

### Anterior thalamus to cortex

#### Retrosplenial cortex afferents (retrograde tracer injections)

**Fig. 7:**
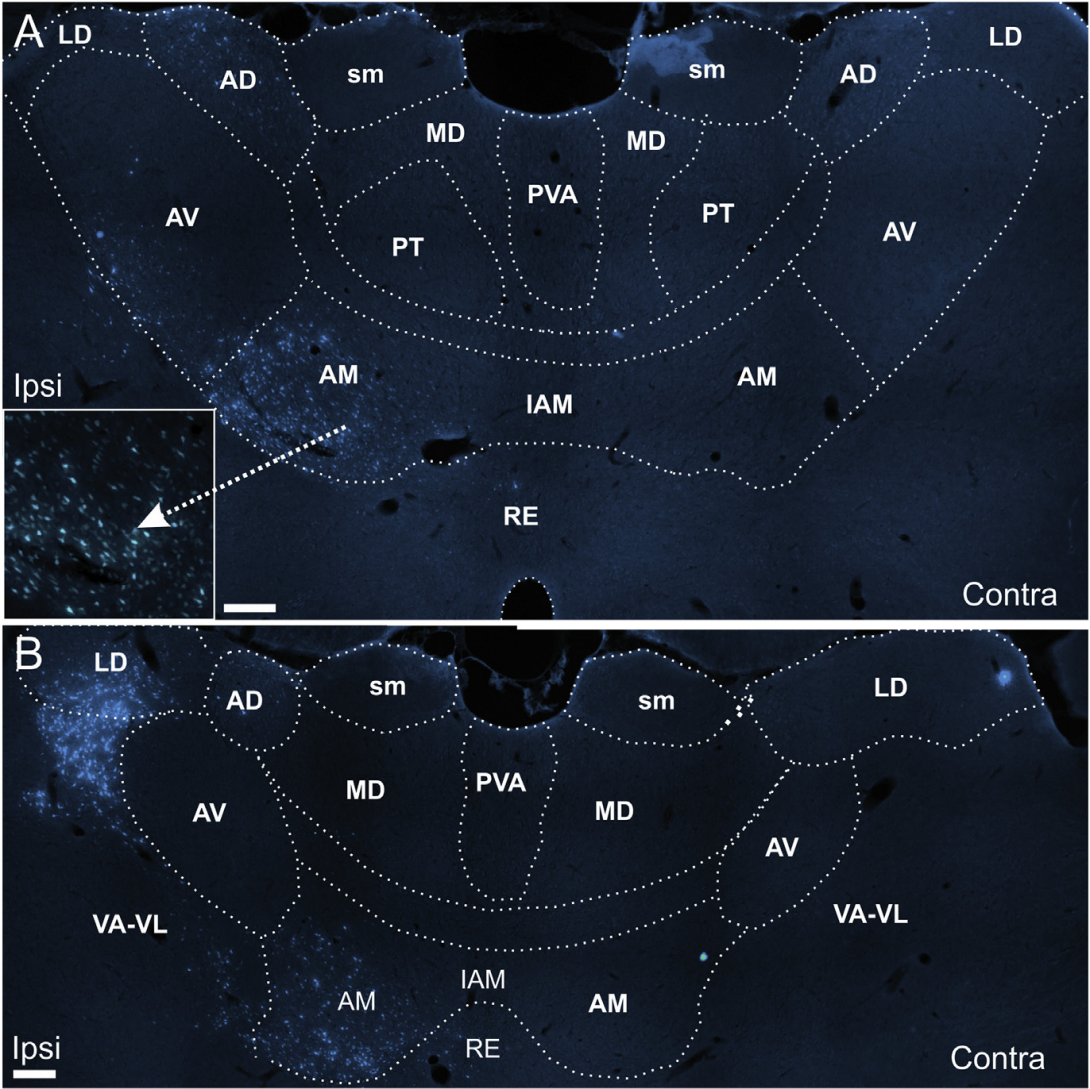
Fluorescence photomicrographs of retrogradely labeled cells in the anterior thalamic nuclei resulting from a fast blue injection in dysgranular retrosplenial cortex (case 64#3). The injection site is shown in Fig.1E. (A) Photomicrograph showing dense AM, and weaker AV and AD labeling restricted to the ipsilateral hemisphere. (B) Photomicrograph showing ipsilateral cell labeling in LD and AM, with no cell labeling in the contralateral hemisphere. Scale bars = 200 μm. [Note, the two, contralateral fluorescent signals (one in AM, one in LD) are not from neurons.]

**Fig. 8:**
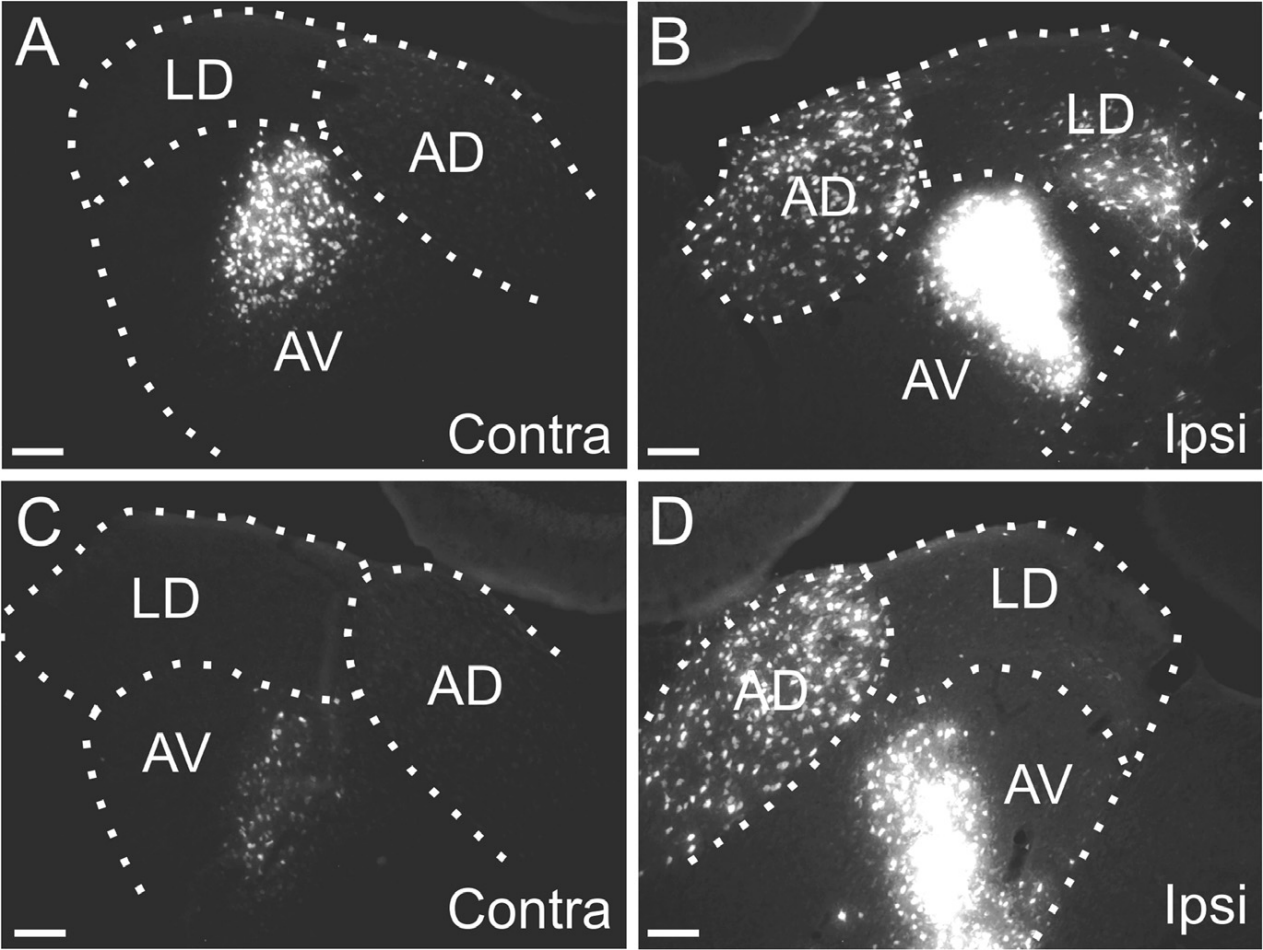
Grayscale fluorescence photomicrographs illustrating the distribution of retrograde label in the anterior thalamic nuclei after a fast blue injection in the retrosplenial cortex. Injection sites are shown in Fig.1C and D. (A, B) Bilateral AV label within the anterior thalamic nuclei in the contralateral (A) and ipsilateral (B) hemispheres (case 172#27). (C, D) Bilateral AV label in the anterior thalamic nuclei in the contralateral (C) and ipsilateral (D) hemispheres (case 172#28). In contrast the LD and AD label is only present ipsilateral to the injection (A, B, C, D). Scale bars = 150 µm.

In five cases, FB was injected along the a-p axis of the dysgranular retrosplenial cortex with varying weak involvement of nearby cortical regions (Table 1). In all five cases, ipsilateral label was present in all of the principal anterior thalamic nuclei as well as LD. In three of these cases (64#3, 77#26, 64#6) the thalamic cell labeling was confined to the ipsilateral hemisphere (Fig. 7, injection site in Fig.1E), whereas in the two remaining cases (172#27, 172#28) a restricted patch of cell labeling was also seen in the contralateral hemisphere (Fig. 8, injection site in Fig.1C,D). In both cases, this contralateral label consisted of a small cluster of retrogradely labeled cells in a restricted caudal-dorsal portion of the AV nucleus (Fig. 8). The patch was very small in a-p extent and resembled a smaller, mirror image of the AV plexus in the ipsilateral hemisphere.

We also analyzed the retrograde transport of BDA (cases 186#4; 188#5; 187#9, described above) as these were all primarily centered in the granular portion of the retrosplenial cortex (while the FB injections were centered in the dysgranular portion). In all three BDA cases the retrograde label was exclusively in the ipsilateral hemisphere, with labeled cells in the AD and AV nuclei, as well as in AM in one case (187#9).

#### Medial frontal cortex afferents (retrograde tracer injections)

In four cases, FB injections were positioned along the a-p axis of the medial frontal cortex (186#4, 187#9, 187#3, 188#3). In three of these cases the tracer injections involved both the anterior cingulate and the prelimbic cortices, primarily involving layers III-V. In the fourth case (188#3) the injection was restricted to the anterior cingulate cortex and was centered in layer V.

**Fig. 9:**
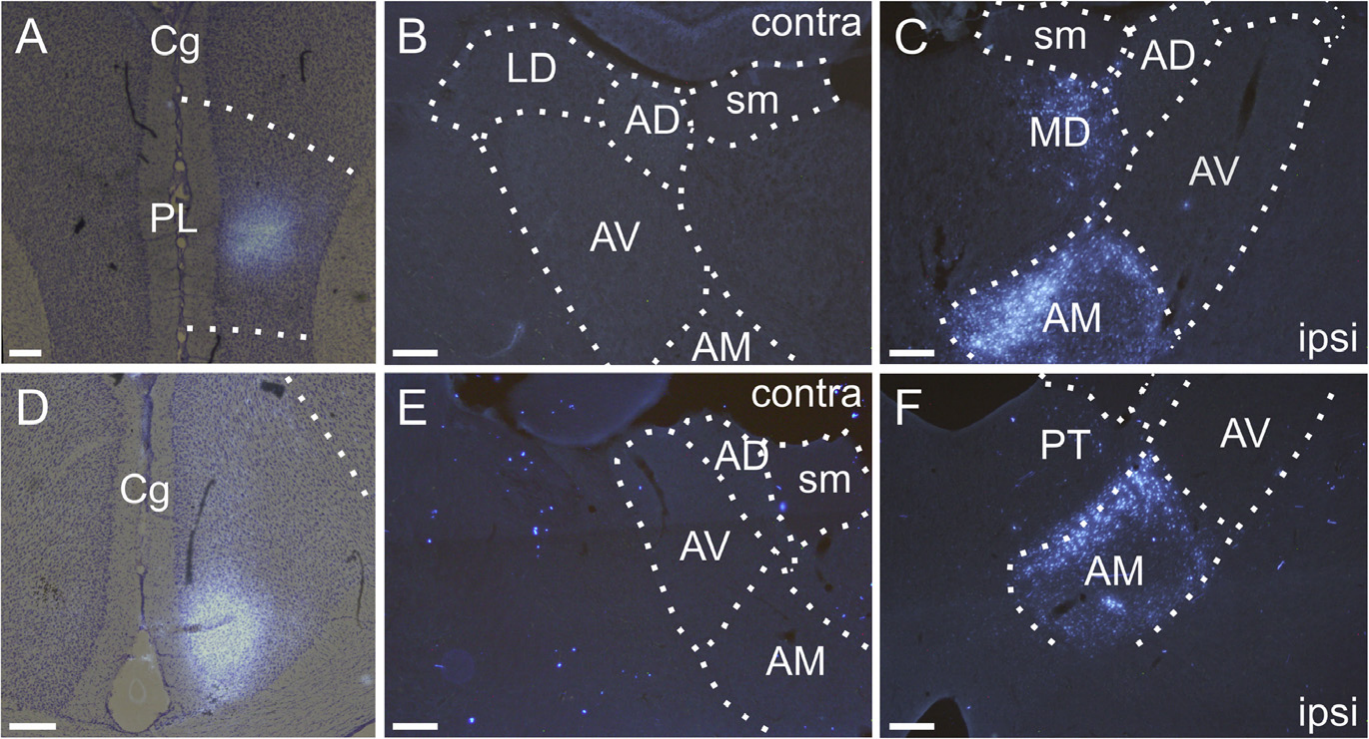
Distribution of retrograde label following fast blue injections in both prelimbic and anterior cingulate cortices (case 187#9, A; case 187#3, D). Both images consist of a low-resolution fluorescence photomicrograph with its corresponding cresyl stained section. (B, C) Fluorescence photomicrographs of the contralateral (B) and ipsilateral (C) anterior thalamic nuclei in case 187#9. (E, F) Fluorescence photomicrographs of the contralateral (E) and ipsilateral (F) anterior thalamic nuclei in case 187#3. Scale bars = 200 µm.

In all four cases, an almost identical labeling pattern was observed. In no case was label observed in the ATN or LD contralateral to the injection site. Label in ATN was largely confined to the ipsilateral AM nucleus (Fig.9B,C,E,F), where a dense region of retrogradely labeled cells was seen primarily around the edge of the nucleus, i.e., avoiding the center of AM as seen on coronal sections. Virtually no label was observed in the ipsilateral AD or AV nuclei. In addition, retrogradely labeled cells were sometimes observed in a confined dorsomedial portion of the ipsilateral LD nucleus (not shown). Consistent with these four cases, when WGA-HRP was centered in the rostral prelimbic cortex (two cases described in the section above, see Table 1), retrograde label was seen only in ipsilateral AM.

#### Subiculum afferents (retrograde tracer injections)

All three injections described above in the section for anterograde subicular transport also resulted in retrogradely labeled cells in the anterior thalamus. In one case with WGA-HRP injections in the subiculum (82#2), dense retrograde label was seen in AD but this label was only ipsilateral. Also in this case, with ventral subiculum and CA1 involvement (82#2), additional cell labeling was seen in the ipsilateral AV and LD nuclei. In the two BDA injections (182#3, 182#4) located in the distal subiculum, only ipsilateral label was seen in LD (both cases) or AD (one case). No label (ipsilateral or contralateral) was seen in AM or AV in these two BDA cases.

#### Postsubiculum afferents (retrograde tracer injections)

A large deposit of the retrograde tracer FG covered the postsubiculum, the secondary visual cortex, and a lateral portion of dysgranular retrosplenial cortex (196#18). Dense cell labeling was present in ipsilateral LD, AV and AD. More moderate label was present in the ipsilateral AM nucleus. No contralateral label was observed in any of these nuclei.

#### Anterior thalamic nuclei – anterograde transport

Three injections of anterograde tracers (Table 1) were centered in the ATN (28#8, 37#4, 183#12). In all cases the injection sites were deliberately large as we intended to fill a large proportion of the ATN with tracer. Consequently, the injection sites involved portions of adjacent thalamic nuclei as well as (in two cases) very restricted parts of the hippocampal formation (Table 1). Despite the injection size, in none of the three cases did we observe fibers in the contralateral cortex or contralateral hippocampal formation.

**Fig. 10:**
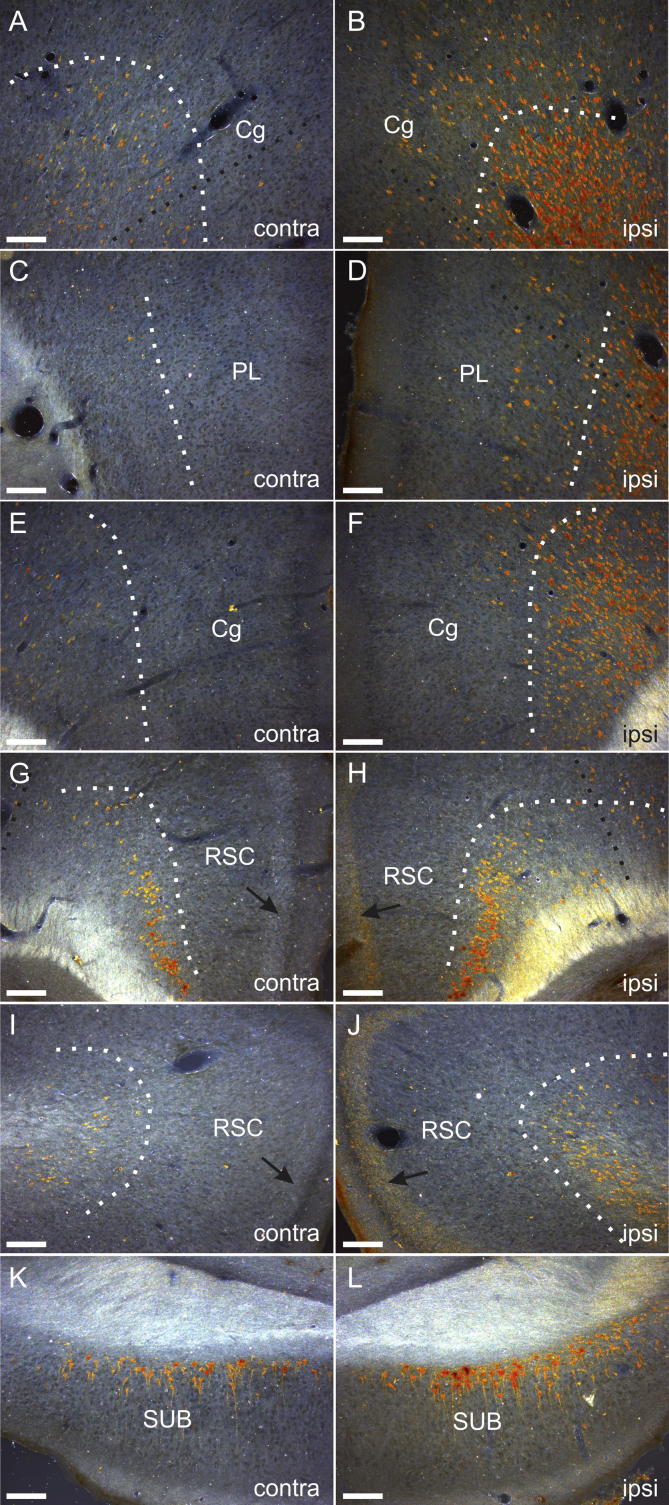
Photomicrographs illustrating the distribution of cell and fiber labeling resulting from a WGA-HRP injection centered in the ATN (case 28#8). The label is shown in ipsilateral (B, D, F, H, J, L) and corresponding contralateral (A, C, E, G, I, K) hemispheres in the rostral anterior cingulate cortex (A, B), prelimbic cortex (C, D), the caudal anterior cingulate cortex (E, F), rostral retrosplenial cortex (G, H) and caudal retrosplenial cortex (I, J), as well as the subiculum (K, L). The white dots indicate the border between layers V and VI. The arrows in H and J point to the superficial fiber plexus in the ipsilateral retrosplenial cortex (anterograde label), which contrasts with the lack of anterograde label in the same layers in the corresponding contralateral hemisphere. The dense color at the pial surface shown in photomicrographs D, J, K, L is artifactual and does not illustrate positive label. Scale bars = 150 µm.

**Fig. 11:**
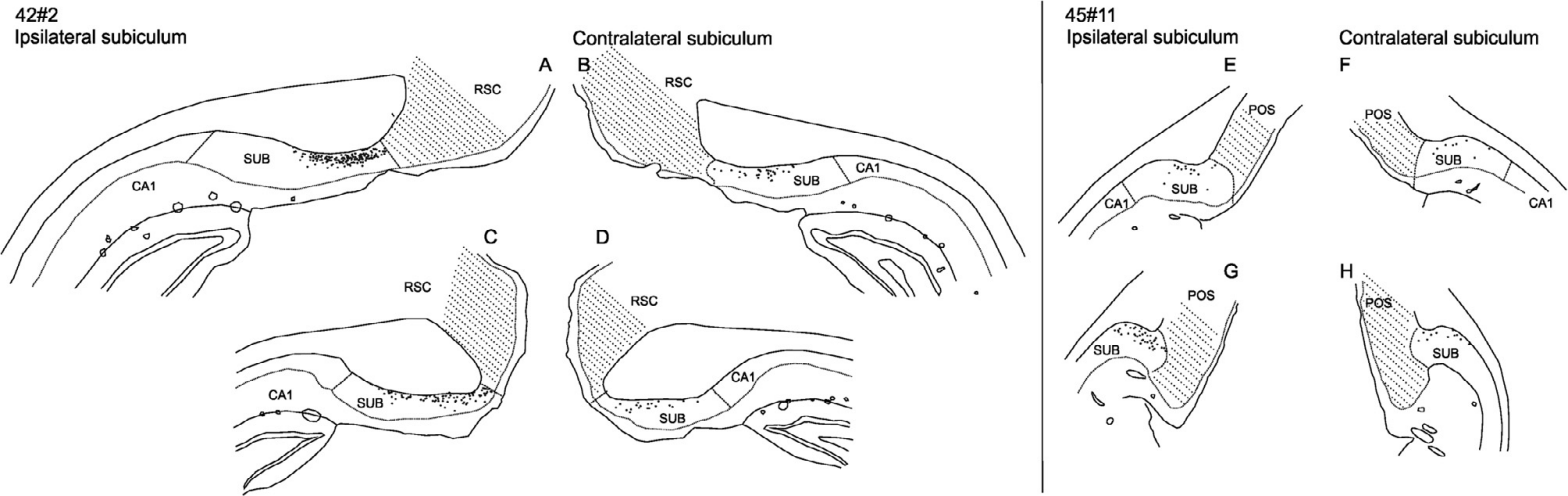
Line drawings of retrograde labeled cells in the subiculum in cases 42#2 (DY, AV nucleus injection) and 45#11 (FB (AM + AV nucleus injection). The injection sites are plotted in Fig.4A-D (case 42#2) and Fig.4L-M (case 45#11). Importantly, note that retrograde labeled cells are plotted only in the subiculum. For postsubiculum and retrosplenial data see Fig. 5 and Fig. 6. (A-D) Plot of retrograde labeled DY cells in the ipsilateral (A, C) and contralateral (B, D) hemisphere in case 42#2. (E-H) Plot of retrograde labeled FB cells in the ipsilateral (E, G) and contralateral (F, H) hemisphere in case 45#11.

Dense ipsilateral terminal labeling was present in the retrosplenial cortex (primarily the granular portion), subiculum and postsubiculum (case 28#8) (Fig. 10), with only weak fiber labeling in layer I of the caudal portion of anterior cingulate cortex in two cases with WGA-HRP injections (37#4, 28#8). In the third case however (183#12, BDA injection), dense terminal fiber labeling was also observed in layer I of both the prelimbic and anterior cingulate cortices.

## Discussion

### Anatomical and technical considerations

Both anterograde and retrograde tracers revealed the patterns of ipsilateral and contralateral connections between the anterior thalamic nuclei and cortex. To counteract the problem of confining a tracer within a specific thalamic nucleus, complementary tracer injections were placed in key cortical and hippocampal sites. The overall goal was to test the general principle that ipsilateral anterior thalamic efferents contrast with bilateral cortical afferents. The anterior thalamic nuclei were the specific focus as they form a key node in Papez circuit, a set of connections that provide a flow of information from the hippocampus to the thalamus and back to the hippocampal formation via the cingulate gyrus (Papez, 1937, Shah et al., 2012). Reflecting these connections, the ATN are vital for aspects of memory (Aggleton and Brown, 1999, Carlesimo et al., 2011).

While previous tracing studies have shown that the ATN receive some bilateral cortical and hippocampal inputs (van Groen and Wyss, 1990b, van Groen and Wyss, 1992a, Shibata, 1998, Van Groen and Wyss, 2003, Shibata and Naito, 2005), the present study sought to compare the details of the corresponding ipsilateral and contralateral connections, including an analysis of both ATN *afferents* and *efferents*. The results of these analyses, as described in the following sections, demonstrate a heterogeneous pattern of crossed inputs to the ATN (including the adjacent LD) nuclei, with hemispheric differences in the laminar origin of the corticothalamic projections, set alongside differences in the relative strengths of the crossed inputs to AM and AV.

**Fig. 12:**
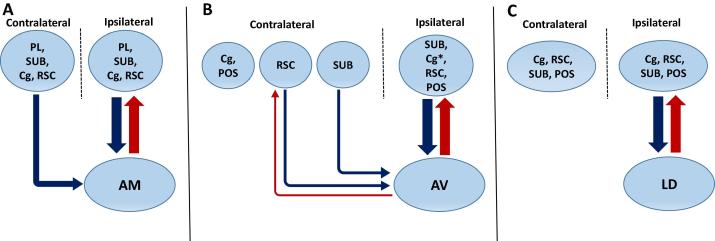
Diagram illustrating the major direct interhemispheric connections that link the AM (A), AV (B) and LD (C) nuclei with neocortex and the hippocampal formation. Thalamocortical projections are shown in red while corticothalamic pathways are shown in blue. For the AV and AM nuclei, the dominant rule is that of bilateral cortical inputs combined with unilateral thalamic outputs to the same cortical sites. Exceptions are a bilateral AV projection to the retrosplenial cortex and a unilateral cingulate projection to AV. In contrast, for the LD nucleus all connections are ipsilateral, as is the case for all postsubiculum connections with the ATN. Arrow size provides an approximate guide to the strength of the connections. Note that Cg in (B) has an asterisk to indicate that this region receives only a relatively weak or restricted input from AV. Overall, our data indicate that the relative numbers of contralateral inputs to AV are less than the corresponding contralateral inputs to AM. Note, the AV input from prelimbic cortex is not shown in (B) as, in our cases, the ipsilateral projection was too weak to meaningfully consider the status of any contralateral projections.

With few exceptions, both AM and AV have dense *ipsilateral* thalamic outputs to the cortex, combined with *bilateral* inputs from the cortex (Fig. 12). This same asymmetric pattern of ipsilateral thalamic efferents and bilateral afferents also applied to IAM. The crossed thalamic inputs vary in their density from cortical site to site, with both AV and, especially, AM receiving substantial bilateral inputs from the subiculum and retrosplenial cortex. In addition, AM also receives a dense bilateral projection from both the prelimbic and anterior cingulate cortices, while the corresponding crossed anterior cingulate projections to AV remain extremely sparse. Furthermore, as the prelimbic input to AV was extremely weak in our dataset, we could not conclude whether a lighter contralateral component was present.

Despite the overwhelming proportion of ANT projections to ipsilateral cortical targets, evidence was found for a light, crossed projection from AV to the dysgranular retrosplenial cortex. This crossed projection originated from a very restricted portion of AV (cell labeling was observed in no more than two sections), presumably explaining why the anterograde tracer injections in ATN did not show contralateral fiber labeling. It can also be assumed that this crossed projection only terminates in a limited portion of the retrosplenial cortex as it was only evident in some of the retrograde tracer cases.

Our analyses also revealed two regions (the postsubiculum and LD) with almost exclusively ipsilateral input and output connections. First, in contrast to the many cortical areas examined, the projections from the postsubiculum remained ipsilateral with respect to their thalamic targets. Likewise, the corresponding reciprocal connections were ipsilateral. Second, unlike AM and AV, the laterodorsal nucleus appears to receive only ipsilateral projections from the subiculum and postsubiculum, as well as from the anterior cingulate and retrosplenial cortices. All of these inputs are reciprocated by projections from LD, which again remain ipsilateral. A caveat regarding the findings for LD is that while the various anterograde tracer cases gave the picture described above, the retrograde tracer injections involving LD led to some limited contralateral label in the retrosplenial and anterior cingulate cortices. Given the difficulty of ensuring that no retrograde tracer injection spread from LD to LP and VA/VL, while also noting previous reports that both the retrosplenial and the anterior cingulate cortical projections to LD remain exclusively ipsilateral (Shibata, 2000, Shibata and Naito, 2005), the overall conclusion is that these LD inputs are solely ipsilateral.

While we did not confine retrograde tracers within the AD nucleus, in some cases we could see light anterograde fiber labeling in this same nucleus. This AD label was essentially restricted to the ipsilateral hemisphere, with just a few individual fibers in a minority of cases in the contralateral side. These observations agree with previous reports (Seki and Zyo, 1984, Shibata and Naito, 2005) that the cortical inputs to AD are overwhelmingly ipsilateral (see below). In a number of cases with thalamic injections, we also observed retrograde cell labeling in the secondary motor cortex (M2). We did not systematically analyze this projection but it was evident that at least the M2 projection to AM is bilateral (Fig6C-F, see also Shibata and Naito, 2005, Alloway et al., 2008).

The quantitative analyses consistently indicated that AM receives more contralateral cortical inputs than AV. This difference was apparent in both the subiculum and the retrosplenial cortex, two areas that provide bilateral input to both thalamic nuclei. Furthermore, rostral to retrosplenial cortex, the cingulate cortex projections to AV are almost exclusively ipsilateral, contrasting with the dense contralateral projections from the anterior cingulate cortex to the AM nucleus.

The ipsilateral and contralateral cortical projections to the ATN did not always show quite the same profile of laminar origin. It has already been observed that the ipsilateral ATN projections originate from across retrosplenial layer VI while the corresponding, contralateral projections arise from more superficial levels within layer VI (Sripanidkulchai and Wyss, 1987). We again found dense ipsilateral cortical projections to AV and AM predominantly from layer VI, but also observed a much lighter contribution from layer V. Meanwhile, the corresponding crossed projections appear to arise exclusively from layer VI. This laminar organization, i.e., additional layer V cells on the ipsilateral side, agrees with what has previously been described for motor cortex projections to the rostral thalamus (Alloway et al., 2008). A potential concern is that we only observed sparse ipsilateral labeling in layer V and, overall, the contralateral labeling was typically weaker than the ipsilateral label, hence, potentially explaining the apparent lack of layer V contralateral label. However, as layer V label was observed exclusively ipsilateral in those portions of cingulate cortex with relatively dense contralateral label, this explanation seems unlikely.

In primates, the layer V component of the frontal corticothalamic projection to AM appears much more numerous, with layer V neurons constituting approximately a third of all projection cells (Xiao and Barbas, 2002, Xiao et al., 2009). This result contrasts with our finding of a much weaker layer V component in rats. We did, however, notice that in a number of cases where the retrograde tracer deposit extended into immediately adjacent parts of other thalamic nuclei (e.g., the ventral anterior nucleus) an appreciable layer V component was now evident, being more comparable to that seen in primates (see cell labeling in Fig. 10). This finding indicates that frontal thalamocortical connections in rats include many projections from layer V, although few reach the AM nucleus.

Previous studies seemingly disagree on whether the anterior cingulate projections to AV are ipsilateral (Shibata and Naito, 2005) or bilateral (Seki and Zyo, 1984). Our combined anterograde and retrograde tracing data strongly support the notion that this pathway is ipsilateral to AV but bilateral to AM. Meanwhile, the prelimbic projections to AM have also been described as predominantly ipsilateral (Shibata and Naito, 2005), yet we consistently found quite evident bilateral projections (although the contralateral component is considerably weaker than that in the nearby anterior cingulate cortex).

Apart from the already mentioned differences in the laminar origin of corticothalamic projections, the present findings indicate only minor species differences between rats and primates. The overall pattern in nonhuman primates is again of bilateral anterior thalamic inputs and solely ipsilateral projections from AV and LD (Amaral and Cowan, 1980, Preuss and Goldman-Rakic, 1987, Vogt et al., 1987, Dermon and Barbas, 1994). Among the ATN, the primate AM stands out as the principal recipient of crossed cortical inputs (Aggleton et al., 1986, Aggleton et al., 2014, Preuss and Goldman-Rakic, 1987), as also now observed in the rat. In primates, AM is also the only consistent source of crossed efferents from the ATN (Preuss and Goldman-Rakic, 1987, Dermon and Barbas, 1994) yet in the rat, the only crossed thalamic projections to the cortex we observed originated from AV.

### Functional considerations

The asymmetric pattern of crossed corticothalamic connections in the rat and monkey informs the finding of simultaneous bilateral activation of the thalamus and cortex in resting state fMRI (Zhang et al., 2008). These authors concluded that the inter-hemispheric thalamocortical co-activation did not fit known monosynaptic connectivity between the cortex and the thalamus, being interpreted as mediated either by commissural connections or by the reticular thalamic nucleus (Zhang et al., 2008). Our data, and others (see above), suggest a more direct explanation, namely that bilateral synchronous thalamocortical activation involves the crossed connections from the cortex to the thalamus.

At the same time, the ipsilateral nature of the thalamic efferents helps to explain why the disruptive effects of unilateral ATN lesions on retrosplenial activity in humans (Jones et al., 2011) and rats (Jenkins et al., 2002) are far more evident in the ipsilateral hemisphere compared to the contralateral hemisphere. Furthermore, the preponderance of ipsilateral routes from the anterior thalamic nuclei to the hippocampal formation not only helps to explain why crossed unilateral lesions between these two structures can be so disruptive to tests of spatial memory (Warburton et al., 2001, Henry et al., 2004) but also indicates that a part of the disconnection effect is from the thalamus to the hippocampus (Warburton and Aggleton, 1999, Dillingham et al., 2015). At the same time, the evidence of gradual improvement on tasks, such as T-maze alternation after crossed (disconnection) lesions (Warburton et al., 2001), may well reflect a growing contribution from the crossed hippocampal efferents to the ATN.

The functional consequences of the thalamocortical connection patterns stem from the physiological properties of the neurons in question. An important distinction is between tonic and bursting firing properties, as well as the concept of “driver” and “modulatory” inputs to thalamic nuclei (Sherman and Guillery, 1998, Sherman and Guillery, 2002, Sherman, 2007, Sherman, 2012, Theyel et al., 2010). Thalamic relay cells respond to excitatory inputs in either bursting or tonic response modes, while the nature of the information transmitted by thalamocortical pathways depends on the type of thalamic response (Sherman, 1996, Sherman, 2001, Reinagel et al., 1999). Whether a thalamic relay cell responds in the burst or tonic mode is thought to depend on modulatory feedback from neocortical layer VI, whereas feedforward corticothalamic communications are thought to be transmitted via the more modest corticothalamic input from layer V (Li et al., 2003, Reichova and Sherman, 2004, Van Horn and Sherman, 2004, Sherman, 2007, Theyel et al., 2010, Varela, 2014). In this context, our finding that the crossed projections to the ATN from the neocortex originate almost exclusively from layer VI, suggests an interpretation in terms of feedforward-feedback mechanisms according to which predominantly ipsilateral feedforward thalamic efferents are reciprocated by bilateral feedback projections with a predominantly modulatory function. Accordingly, although bilateral corticothalamic projections from layer VI are abundant, they might be “modulating” rather than “driving” the thalamus. Indeed, one feature of such modulatory projections (layer VI) is that they outnumber driving inputs (Sherman, 2016). Consequently, this latter function may involve the less numerous layer V projections (Sherman, 2016), which we found to be ipsilateral. These cell-type distinctions become relevant to Papez ‘circuit’ as the interactions between the ATN and cingulate cortices are reciprocal, rather than simply efferent from the thalamus, as implied by the concept of a circuit.

Two regions, the postsubiculum and LD, seemingly possess almost exclusively ipsilateral afferents and efferents. Both sites are part of the head-direction system (Mizumori and Williams, 1993, Taube, 2007), i.e., they contain cells that fire when the animal’s head is facing a specific direction. Other key regions in this system include AD (Taube, 1995), which occupies a pivotal position as it transmits head-direction information from the lateral mammillary bodies (Blair et al., 1998, Stackman and Taube, 1998) to the neocortex and hippocampal formation (Taube et al., 1990b, Taube et al., 1990a, Goodridge and Taube, 1997, Cho and Sharp, 2001).

It is natural to speculate that the ipsilateral nature of the postsubiculum and LD nucleus connections is related to their key roles in rodent navigation. A logical extension is that AD should also show the same ipsilateral connectivity pattern. Since we often observed relatively sparse inputs to AD it is difficult to be definitive on this issue. However, as mentioned, when more dense fiber labeling was present in AD, this label was always ipsilateral. These observations accord with previous studies that have described the AD input from both the anterior cingulate and retrosplenial cortices, as well as from the postsubiculum, as being ipsilateral (Seki and Zyo, 1984, Shibata and Naito, 2005). A well-known exception concerns the bilateral inputs to AD from the lateral mammillary nucleus (Shibata, 1992), which have been characterized as “drivers” (Petrof and Sherman, 2009). These inputs from the mammillary bodies differ from the cortical connections of AD as they are unidirectional, i.e., not reciprocated (Seki and Zyo, 1984, Shibata, 1992). It may be that the head direction system has unique constraints that relate to its use of interoceptive (e.g., vestibular) and exteroceptive (e.g., visual) cues in signaling direction and aiding path integration (Taube, 2007), which select against reciprocal, crossed cortical connections.

For AM and AV, the dominant rule is one of bilateral cortical inputs combined with unilateral thalamic outputs to the same cortical sites (Fig. 12). Furthermore, it is AM that receives the greater proportion of crossed inputs, a difference that is especially marked for the inputs from the anterior cingulate cortex. There are, in addition, plentiful callosal connections linking corresponding neocortical sites. In contrast, thalamic nuclei are typically not directly interconnected across the midline, although the reticular thalamic nucleus might provide an indirect route (Raos and Bentivoglio, 1993). Accordingly, the bilateral structure of corticothalamic feedback projections could be a functional consequence of the lack of bilateral intrinsic thalamic connectivity. That is, as information is not integrated directly between the two hemispheres at the level of the thalamic nuclei, interhemispheric integration is achieved by corticothalamic pathways. The potential for this integration is further enhanced as transynaptic tracing data shows how cells in layer II of the retrosplenial cortex, which is the source of its interhemispheric retrosplenial – retrosplenial connections, also project to those layer VI cells that then project to the anterior thalamus (Amin et al., 2010). Consequently, the unilateral thalamic input to cortex might be sufficient to ensure bilateral integration as required, given the multiple routes to integrate signals among the cortical hemispheres.

## Figures and Tables

**Table 1:** Table of all anterograde and retrograde tracer injections analyzed. Layers indicated in the third column indicate the center of the tracer deposit and do not exclude potential involvement of other layers. Those regions and layers in parenthesis indicate weak involvement in the injection site

Case #	Target (tracer)	Injection site
*Retrograde injections in cortex*		
187#9	FB	PL/Cg, layers III/V
187#3	FB	PL/Cg, layers III/V
186#4	FB	PL/Cg (M2), layers III/V
188#3	FB	Cg, layer V
64#6	FB	RSC, layers I-VI
172#27	FB	RSC, layers I-V (M2, V2)
172#28	FB	RSC, layers I-V (M2)
77#26	FB	RSC, layers I-V (V2)
64#3	FB	RSC, layers I-V (VI), M2/V2
196#18	FG	PoS/V2/RSC

*Anterograde injections in cortex*		
199#9	BDA (3kD)	Cg, layers II-VI (M2)
199#10	BDA (3kD)	Cg, layers II-VI (M2)
199#11	BDA (10kD)	Cg, layers III-VI
187#9	BDA (3kD)	RSC, layers V/VI
186#4	BDA (3kD)	RSC, layers II-V
188#5	BDA (3kD)	RSC, layer V
182#3	BDA (3kD)	SUB (V1/2/PoS)
182#4	BDA (3kD)	SUB (V1/2)

*WGA-HRP injections in cortex*		
88#1	WGA-HRP	PL, layers V/VI
88#2	WGA-HRP	PL, layers III/V
82#2	WGA-HRP	SUB/HPC

*Retrograde injections in the anteroventral and anteromedial thalamic nuclei*		
88#5	FB	AV
42#2	DY	AV
198#2	FB	AM (IAD, HPC)
41#5	DY	AM
45#11	FB	AV/AM

*Retrograde injections centered in, but not restricted to, the anterior thalamic nuclei*		
88#6	FB	AV/VA
41#5	FB	AV/AD (AM, VA)
45#11	DY	AM (PT)
42#2	FB	AM (PT)
191#9	FB	AV (AD, VL, AM)
198#4	ctb	AV (AM, PT)

*Retrograde injections centered in, but not restricted to, the laterodorsal thalamic nucleus*		
191#10	FB	LD (LP, HPC)
196#19	FG	LD (VA-VL)

*WGA-HRP and BDA injections centered in, but not restricted to, specific anterior thalamic nuclei*		
28#8	WGA-HRP	AV/VA/AD/AM (HPC)
37#4	WGA-HRP	AV (AD, AM, HPC)
183#12	BDA (3kD)	AV (AD, AM, VA, PT)
